# Long term efficacy and safety of rivaroxaban plus cilostazol in the treatment of critical ischemia of the lower limbs in a frail, elderly patient with non valvular atrial fibrillation

**DOI:** 10.1186/s40780-020-00173-9

**Published:** 2020-08-03

**Authors:** Antonio Trani, Pietro Benedetto, Ferdinando Di Leo, Angela Baiano, Andrea Esposito, Danilo Menna, Arianna Allegretti, Pierluigi Antonino Cappiello, Domenico Dell’Edera

**Affiliations:** 1 Vascular and Endovascular Surgery Unit, Cardiovascular Department, San Carlo Hospital Potenza, via Potito Petrone, 85100 Potenza, Italy; 2 Cytogenetic and Molecular Genetics Unit, “Madonna delle Grazie” Hospital, 75100 Matera, Italy

**Keywords:** Cilostazol, Critical lower limb ischemia, Elderly, Non valvular atrial fibrillation, Rivaroxaban

## Abstract

**Background:**

Many patients with critical lower limb ischemia are not eligible for revascularization procedures. Still, given the emerging role of both platelet and coagulation activation in the formation of arterial thrombi, they may benefit from the novel anticoagulant and antiplatelet drugs.

**Case presentation:**

We describe the case of a male with critical lower limb ischemia complicated by older age, frailty, polymorbidity and non valvular atrial fibrillation, who was deemed as non eligible for surgery. The patient was successfully treated with the combination of rivaroxaban and cilostazol, and the clinical benefit was maintained throughout 32 months, with no occurrence of major or minor hemorrhagic or thrombotic events.

**Conclusions:**

To our knowledge, this is the first report on the efficacy and safety of such a combination therapy in critical lower limb ischemia. In a clinical setting in which alternative pharmacological approaches are urgently needed, the association of rivaroxaban and cilostazol warrants further investigations.

## Background

Currently, the treatment of critical lower limb ischemia, a sequelae of peripheral arterial disease (PAD), remains a challenge [1]. Despite the progress in revascularization procedures, many patients present multiple comorbidities, frailty (i.e., age > 80 years, body weight < 60 kg and HAS-BLED ≥3), and severe, diffuse lower limb atherosclerosis, which hamper their eligibility to surgical, endovascular or hybrid procedures [1, 2]. Yet, they may benefit from the novel anticoagulant and vasoactive antiplatelet drugs [3, 4], as both platelet and coagulation activation participate in the formation of arterial thrombi [4]. Thus, in patients with arterial diseases, the indication for antiplatelet therapy (recommended in first line) does not exclude anticoagulation [3–5]; rather, the association may be even superior to the antiaggregant agent alone, as shown by the recent COMPASS trial [5]. Indeed, this phase III study, which was prematurely stopped for overwhelming efficacy when only one half of the primary end-point events had occurred [6], showed the superiority of rivaroxaban plus aspirin, compared to aspirin alone, in reducing the risk of cardiovascular death, stroke, or myocardial infarction in patients with a history of stable atherosclerotic vascular disease. Rivaroxaban belongs to the class of the direct oral anticoagulants (DOACs), currently considered an advantageous alternative to the standard oral anticoagulants (i.e., the vitamin K antagonists, Vitamin K antagonists). Notably, DOACs, and, in particular, rivaroxaban, have yielded an acceptable benefit/risk ratio in patients with different venous and arterial thromboembolic diseases even when antiaggregants were required [7, 8].

Among antiplatelet agents, cilostazol, a potent phosphodiesterase type III selective inhibitor, is the only vasoactive and antiproliferative drug with documented evidence (grade IA) in the treatment of PAD when associated to traditional antiplatelet drugs [9]. Cilostazol has been shown to improve the walking distance in patients with intermittent claudication (Fontaine’s stage IIA or IIB) [10], and is now recommended as the first choice in lower extremity PAD up to advanced stage IIB. Moreover, it consistently reduced restenosis/reocclusion after surgical and endovascular interventions in patients with critical ischemia, providing middle long term efficacy and decreased risk of hemorrhagic complications [11, 12]. In patients undergoing femoropopliteal stenting, cilostazol significantly reduced the rate of restenosis/reocclusion and improved the primary patency rate, especially in subjects at high risk of in stent restenosis [13]. Moreover, it is the only agent that demonstrates, in randomized controlled trials, adequate efficacy in decreasing the occurrence of restenosis/reocclusion after coronary or peripheral revascularization procedures [14].

Is known that in elderly patients, the increase in the number of antiaggregant and anticoagulant drugs increases the rate of hemorrhagic events.

Some important studies document how this risk is reduced with the use of cilostazol provided that the therapy is personalized and the dosages in high risk cases are the minimum expected (50 mg twice daily for cilostazole and 15 mg daily for rivaroxaban in patients with CAD and PAD and non-valvular atrial fibrillation) [15–20].

Moreover, cilostazole is not a traditional antiaggregant but is now considered by the scientific community as a drug “also antiaggregant” and the only one with strong evidence of anti-inflammatory, anti-restenosis and with current indications in the treatment of brain hemorrhage.

Given that antiaggregants are not contraindicated in patients already on DOACs because of non valvular atrial fibrillation (NVAF) or venous thromboembolic disease, and in those with carotid, coronary or peripheral stents, rivaroxaban and cilostazol might be good candidates for the treatment of patients with critical limb ischemia not eligible for surgery [21].

COMPASS study [22, 23] and VOYAGER-PAD study [24], stress the importance the association rivaroxaban with ASA in the prevention of ischemic limb events and in the substantial reduction of the rate of major amputations (about 70%).

Here, we report the efficacy and safety of rivaroxaban plus cilostazol in a case of critical lower limb ischemia not suitable for revascularization, complicated by NVAF, frailty, older age and several comorbidities. Data for 32 months of follow-up are presented.

This association has shown that low dosages of both molecules (15 mg/day of rivaroxaban and 50 mg/2/day of cilostazole), it resulted in limb salvage and prevented death from acute heart failure. The patient died at the age of 88 following viral pneumoni.

## Case report

In January 2015, an 86-year-old male was admitted to our Hospital for critical ischemia of lower limbs characterized by rest pain, cyanosis on feet, acral ulcers, hypothermia below the knee socks and partial sensory-motor peripheral neuropathy. The patient, a former smoker, presented with frailty and multiple comorbidities, including multi-infarct dementia, previous recurrent transient ischemic attacks (TIA), chronic ischemic heart disease with three-vessel coronary artery disease deemed as non eligible for surgical intervention in 2013, Non-Valve Atrial Fibrillation (NVAF), dyslipidemia, chronic obstructive pulmonary disease (COPD), megaloblastic anemia, prior gastrectomy for gastric ulcer, and mild-to-moderate chronic kidney disease. The patient was not diabetic.

Drug therapy adopted was:

- dyslipidemia being treated with atorvastatin (20 mg/ ay);
- arterial hypetension being treated with zofenopril (30 mg/day), lercanidipine hydrochloride (10 mg / day) and nitroglycerin in transdermal patches (5 mg/day).
- megaloblastic anemia being treated with a supplement based on folate (acid (6S) -5- methyltetrahydrofolic), cyanocobalamin (vitamin B12), pyridoxine (vitamin B6), riboflavin (vitamin B2), betaine and zinc
- chronic obstructive pulmonary disease (BPCO) being treated with glycopyrronium bromide (one inhalation/day).

The patient was unsuitablenot suitable for myocardial (CAD) and peripheral (PAD) revascularization, given the remarkable severity and diffusion of atherosclerotic pathology.

Cholesterol and triglyceride parameters before using atorvastatin were: total cholesterol 250 mg/dl, HDL cholesterol 45 mg/dl, LDL cholesterol 181 mg/dl, triglycerides 120 mg/dl. After use of atorvastatin the values have significantly decreased: total cholesterol 137 mg/dl, HDL cholesterol 57 mg/dl, LDL cholesterol 70 mg/dl and triglycerides 51 mg/dl.

The patient underwent echo color-Doppler and continuous-wave Doppler examination of lower limbs, which revealed multiple steno-obstructive lesions of the iliac, femoropopliteal and tibial segments, and ankle-brachial index (ABI) of approximately 0.3–0.35, bilaterally. Computed tomography (CT) angiography of the aorta, iliac arteries, and lower limb arteries showed extensive fibrocalcific atherosclerotic plaques in the iliac, femoropopliteal and tibial segments, bilaterally, deemed as non-eligible for surgical, endovascular or hybrid procedures. Moreover, CT-angiography of supra aortic vessels and cerebral circulation displayed the presence of multiple severe steno-obstructive lesions of both extra-and intra-cranial arteries.

Treatment with intravenous heparin and antiplatelet drugs (acetylsalicylic acid 100 mg/day and clopidogrel 75 mg/day) was commenced.

After 10 days, the patient was discharged with the indication for anticoagulant and antiplatelet therapy with warfarin (international normalized ratio [INR]: 2–3) and clopidogrel 75 mg/day. As, over the following 3 months, the INR ranged between 2 and 3.5 in only 45% of evaluations, the patient was switched to rivaroxaban 15 mg/day plus cilostazol, which was started at the dose of 150 mg/day and then increased up to 200 mg/day. The choice of the low dose of rivaroxaban (vs. 20 mg/day) was made based on the advanced age, frailty, body weight (< 60 kg) and creatinine clearance (between 15 and 49 ml/min) of the patient [25, 26].

At the following visits, 6, 12, 18, 24 and 30 months after discharge, the clinical and echo color doppler examinations confirmed improved revascularization, with acceptable perfusion of lower limbs, complete healing of acral ulcers present at both admission and discharge, and a clear amelioration of ischemia, disappearance of rest pain (from Fontaine’s stage III to non-advanced stage IIB, with > 100 m of functional autonomy) and improvement of the ABI (from approximately 0.3 [left] and 0.35 [right] before the start of rivaroxaban and cilostazol, to 0.55 [left] and 0.6 [right] afterwards). Such results were maintained over time, in the absence of progression of the other cardiovascular and metabolic diseases and pre-existing conditions.

Approximately 32 months after the start of rivaroxaban plus cilostazol, although several assessments and dose adjustments were required with regard to the medications for concomitant diseases, limb perfusion was still good (the limb was saved), the hematochemical parameters (blood count, coagulation, serum creatinine, and liver function) were stable and no major or minor hemorrhagic or thrombotic events had occurred.

Unfortunately, soon after the last follow-up visit, the patient, who suffered from advanced COPD, died of acute respiratory insufficiency due to severe pulmonary infection. The cause was, in no way, related to the treatment for vascular disease and, indeed, no signs of bleeding were documented.

Furthermore, the patient presents a CHADS2 score (risk of cerebral infarction in patients with atrial fibrillation) equal to 5 and HAS-BLED score (risk of bleeding) equal to 4.

## Discussion

To date, the presence of diffuse steno-obstructive lesions in the arterial occlusive disease of lower limbs, and, in particular, critical lower limb ischemia, makes affected patients frequently non-eligible for traditional surgery procedures or novel molecular and cellular therapies (i.e., growth factors, stem cells, and stem cells from adipose tissue) [27, 28]. This is especially true in the case of frail patients aged > 80 years, who often suffer from multiple diseases. Therefore, alternative pharmacological approaches are urgently needed for the management of these subjects [29].

Here, we report the successful treatment with rivaroxaban plus cilostazol of a patient with critical lower limb ischemia complicated by older age, frailty, and several concomitant cardiovascular and metabolic diseases, including NVAF. Notably, after 32 months, the patient was still in good clinical conditions, without major or minor hemorrhagic or thrombotic events. To the best of our knowledge, this is the first demonstration of the efficacy and safety of such a combination therapy in critical lower limb ischemia. Our results are even more encouraging considering the long follow-up period and the complex clinical conditions of the patient, which are frequently encountered by physicians in their daily practice. Notably, Ozker and coworkers reported on the usefulness of rivaroxaban and cilostazol in a dysfibrinogenemic patient with thrombotic episodes, providing additional evidence that this combination may represent a therapeutic option in certain cases, that warrants further investigations.

As arterial thromboembolic events rely on both pro aggregant and pro thrombotic mechanisms, patients should always be administered anticoagulant and antiplatelet vasoactive agents. In this way, the different steps of primary arterial thrombosis and reocclusion/restenosis can be successfully inhibited. In daily practice, the skilled vascular surgeon/angiologist faces the choice of the most adequate therapeutic strategy based on novel combinations of anticoagulant and vasoactive antiplatelet drugs (possibly feasible also at home). The treatment must maximize the effect on the collateral arterial, muscular and skin circulation, and make these vessel networks more complex and effective to guarantee the most active alternative microcirculation in order to save the limb and prolong patient survival. In this context, a major role may be played by the well documented vasoactive and antiproliferative features of cilostazol, when combined with the antiplatelet and thrombolytic properties of rivaroxaban and its effects on arterial circulation. Such approach allowed to treat the thrombosis of small medium sized arterial vessels (representing the core of collateral circulations) in our case as in previous reports. This hypothesis is corroborated by the complex case of critical lower limb ischemia described here, in which the association of cilostazol and rivaroxaban has positively affected the outcome of artery disease.

At present, the results of the RCT VOYAGER PAD study (ClinicalTrials.gov Identifier: NCT02504216) are eagerly awaited, as it is the only randomized controlled trial (RCT) exploring the efficacy and safety of rivaroxaban in symptomatic PAD following peripheral revascularization procedures of the lower extremities. However, several groups, including ours, have acknowledged the pivotal role of rivaroxaban (sometimes used off-label) as adjuvant therapy after surgical or endovascular procedures in maintaining the efficacy of the intervention and in the salvage of the limb. In our experience, adding cilostazol to rivaroxaban has positively impacted on patient outcome, even after 32 months.

## Conclusion

Success in treating critical limb ischemia is measured by amputation-free survival (AFS). Although patients with critical limb ischemia are revascularized by endovascular or surgical procedures, amputation rates remain high. One year after the onset of critical limb ischemia approximately 25% of patients will have to undergo major limb amputation. While about 35–67% will experience major limb amputation within 4 years. Additionally, early post-operative mortality rates vary from 4 to 22% after major limb amputation [30]. Numerous studies suggest that a polypharmacological approach is needed.

- anticoagulant or antithrombotic associated with anti Xa drugs (rivaroxaban) (fibrillating or non-fibrillating patients).
- antiplatelet agent (acetylsalicylic acid or clopidogrel) in associated coronary artery disease.
- Vasoactive (cilostazol), which plays an important role in the inhibition of proilferative cell replication processes also involved in the evolution of atherosclerosis and in the pathogenesis of restenosis.

These drugs certainly increase bleeding risk but not significantly if used at appropriate dosages. It is clear that this approach must always take into account the benefit/risk ratio and the degree of reversibility-irreversibility of the ischemic framework.

The case report described here supports the association of rivaroxaban and cilostazol as a useful strategy to treat critical lower limb ischemia in patients non-eligible for revascularization procedures and encourages further investigations. In the absence of compelling evidence from RCTs, case reports, which represent the first line of evidence, are crucial to collect data on the efficacy and safety of novel therapeutic strategies.

## Supplementary information

**Additional file 1.**

## Data Availability

All data generated or analyzed during this study are included in this published article.

## Abbreviations

PAD: Peripheral arterial disease
HAS-BLED: Hypertension abnormal renal and liver function stroke bleeding labile inr elderly drugs
DOACs: Direct oral anticoagulants
VKAs: Vitamin K antagonists
NVAF: Valvular atrial fibrillation
COPD: Chronic obstructive pulmonary disease
ABI: Ankle-brachial index
CT: Computed tomography
INR: International normalized ratio
RCT: Randomized controlled trial;
