# Supplementary material for: Long term efficacy and safety of rivaroxaban plus cilostazol in the treatment of critical ischemia of the lower limbs in a frail, elderly patient with non valvular atrial fibrillation
Source: J Pharm Health Care Sci. 2020 Aug 3;6:17. doi: 10.1186/s40780-020-00173-9 (PMC7398073; doi:10.1186/s40780-020-00173-9)
Supplement: Supplementary file 1 — Additional file 1. [file 40780_2020_173_MOESM1_ESM.doc]

**Reviewer #1 - QUESTIONS**

**Question 1.**

Some clinical trials (COMPASS PAD, ATLAS ACS 2-TIMI 51) and Meta-analysis (Yuan, J., BMC Pharmacol Toxicol 19, 2018) have indicated that the safety of combination therapy was still doubtful. The reviewer suggests that the authors should more carefully discuss the safety of the combination (rivaroxaban and cilostazol) therapy in this report.

**Answer 1**

Is known that in elderly patients, the increase in the number of antiaggregant and anticoagulant drugs increases the rate of hemorrhagic events.

some important studies document how this risk is reduced with the use of cilostazol provided that the therapy is personalized and the dosages in high risk cases are the minimum expected (50 mg twice daily for cilostazole and 15 mg daily for rivaroxaban in patients with CAD and PAD and non-valvular atrial fibrillation).

Moreover, cilostazole is not a traditional antiaggregant but is now considered by the scientific community as a drug "also antiaggregant" and the only one with strong evidence of anti-inflammatory, anti-restenosis and with current indications in the treatment of brain hemorrhage.

**REFERENCE**

- Emre Ozker, Figen Atalay, Oyku Gulmez, Bulent Saritas. Treating a Patient of Dysfibrinogenemia with Acute Thromboembolism by Rivaroxaban and Cilostazol. Indian J Hematol Blood Transfus (July-Sept 2017) 33(3):431–433.
- Nikolaos Spinthakis, Mohamed Farag, Bianca Rocca, Diana A. Gorog. More, More, More: Reducing Thrombosis in Acute Coronary Syndromes Beyond Dual Antiplatelet Therapy—Current Data and Future Directions [J Am Heart Assoc.](https://www.ncbi.nlm.nih.gov/pubmed/?term=More%2C+More%2C+More%3A+Reducing+Thrombosis+in+Acute+Coronary+Syndromes+Beyond+Dual+Antiplatelet+Therapy—Current+Data+and+Future+Directions) 2018 Jan 26;7(3).
- Zavgorodnyaya D, Knight TB, Daley MJ, Teixeira PG. [Antithrombotic therapy for postinterventional management of peripheral arterial disease](https://www.ncbi.nlm.nih.gov/pubmed/31930282). Am J Health Syst Pharm. 2020 Feb 7;77(4):269-276.
- [Real J](https://www.ncbi.nlm.nih.gov/pubmed/?term=Real J%5BAuthor%5D&cauthor=true&cauthor_uid=29739318), [Serna MC](https://www.ncbi.nlm.nih.gov/pubmed/?term=Serna MC%5BAuthor%5D&cauthor=true&cauthor_uid=29739318), [Giner-Soriano M](https://www.ncbi.nlm.nih.gov/pubmed/?term=Giner-Soriano M%5BAuthor%5D&cauthor=true&cauthor_uid=29739318), [Forés R](https://www.ncbi.nlm.nih.gov/pubmed/?term=Forés R%5BAuthor%5D&cauthor=true&cauthor_uid=29739318), [Pera G](https://www.ncbi.nlm.nih.gov/pubmed/?term=Pera G%5BAuthor%5D&cauthor=true&cauthor_uid=29739318), [Ribes E](https://www.ncbi.nlm.nih.gov/pubmed/?term=Ribes E%5BAuthor%5D&cauthor=true&cauthor_uid=29739318), [Alzamora M](https://www.ncbi.nlm.nih.gov/pubmed/?term=Alzamora M%5BAuthor%5D&cauthor=true&cauthor_uid=29739318), [Marsal JR](https://www.ncbi.nlm.nih.gov/pubmed/?term=Marsal JR%5BAuthor%5D&cauthor=true&cauthor_uid=29739318), [Heras A](https://www.ncbi.nlm.nih.gov/pubmed/?term=Heras A%5BAuthor%5D&cauthor=true&cauthor_uid=29739318), [Morros R](https://www.ncbi.nlm.nih.gov/pubmed/?term=Morros R%5BAuthor%5D&cauthor=true&cauthor_uid=29739318). Safety of cilostazol in peripheral artery disease: a cohort from a primary healthcare electronic database. [BMC Cardiovasc Disord.](https://www.ncbi.nlm.nih.gov/pubmed/?term=Safety+of+cilostazol+in+peripheral+artery+disease%3A+a+cohort+from+a+primary+healthcare+electronic+database) 2018 May 8;18(1):85.
- [Disdier Moulder MPA](https://www.ncbi.nlm.nih.gov/pubmed/?term=Disdier Moulder MPA%5BAuthor%5D&cauthor=true&cauthor_uid=31825133), [Hendricks AK](https://www.ncbi.nlm.nih.gov/pubmed/?term=Hendricks AK%5BAuthor%5D&cauthor=true&cauthor_uid=31825133), [Ou NN](https://www.ncbi.nlm.nih.gov/pubmed/?term=Ou NN%5BAuthor%5D&cauthor=true&cauthor_uid=31825133). Towards appropriate polypharmacy in older cardiovascular patients: How many medications do I have to take? [Clin Cardiol.](https://www.ncbi.nlm.nih.gov/pubmed/?term=Towards+appropriate+polypharmacy+in+older+cardiovascular+patients%3A+How+many+medications+do+I+have+to+take%3F) 2020 Feb;43(2):137-144.
- [Daimon S](https://www.ncbi.nlm.nih.gov/pubmed/?term=Daimon S%5BAuthor%5D&cauthor=true&cauthor_uid=30088338). Adverse Effect of Antithrombotic Medications on Bleeding Events and Comparison of Antithrombotic Agents in Hemodialysis Patients. [Ther Apher Dial.](https://www.ncbi.nlm.nih.gov/pubmed/?term=Adverse+Effect+of+Antithrombotic+Medications+on+Bleeding+Events+and+Comparison+of+Antithrombotic+Agents+in+Hemodialysis+Patients) 2019 Feb;23(1):32-37.

**Question 2**

The case had non valvular atrial fibrillation and critical lower limb ischemia, and therefore the co-treatment of rivaroxaban and cilostazol seems to be natural when consider the indication of each drug. To avoid the misreading of readers, the authors should clearly mentioned the clinical meanings and importance of this case report.

**Answer 2**

Given that antiaggregants are not contraindicated in patients already on DOACs because of non valvular atrial fibrillation (NVAF) or venous thromboembolic disease, and in those with carotid, coronary or peripheral stents, rivaroxaban and cilostazol might be good candidates for the treatment of patients with critical limb ischemia not eligible for surgery.

COMPASS study and VOYAGER-PAD study, stress the importance the association rivaroxaban with ASA in the prevention of ischemic limb events and in the substantial reduction of the rate of major amputations (about 70%).

Here, we report the efficacy and safety of rivaroxaban plus cilostazol in a case of critical lower limb ischemia not suitable for revascularization, complicated by NVAF, frailty, older age and several comorbidities. Data for 32 months of follow-up are presented.

This association has shown that low dosages of both molecules (15mg/day of rivaroxaban and 50mg/2/day of cilostazole), it resulted in limb salvage and prevented death from acute heart failure. The patient died at the age of 88 following viral pneumoni.

Reference:

- [Tantry U](https://www.ncbi.nlm.nih.gov/pubmed/?term=Tantry U%5BAuthor%5D&cauthor=true&cauthor_uid=32129681), [Cummings C](https://www.ncbi.nlm.nih.gov/pubmed/?term=Cummings C%5BAuthor%5D&cauthor=true&cauthor_uid=32129681), [Mackrell P](https://www.ncbi.nlm.nih.gov/pubmed/?term=Mackrell P%5BAuthor%5D&cauthor=true&cauthor_uid=32129681), [Gonze M](https://www.ncbi.nlm.nih.gov/pubmed/?term=Gonze M%5BAuthor%5D&cauthor=true&cauthor_uid=32129681), [Ulloa K](https://www.ncbi.nlm.nih.gov/pubmed/?term=Ulloa K%5BAuthor%5D&cauthor=true&cauthor_uid=32129681), [Bafford R](https://www.ncbi.nlm.nih.gov/pubmed/?term=Bafford R%5BAuthor%5D&cauthor=true&cauthor_uid=32129681), [Rout A](https://www.ncbi.nlm.nih.gov/pubmed/?term=Rout A%5BAuthor%5D&cauthor=true&cauthor_uid=32129681), [Sukhi A](https://www.ncbi.nlm.nih.gov/pubmed/?term=Sukhi A%5BAuthor%5D&cauthor=true&cauthor_uid=32129681), [Gurbel P](https://www.ncbi.nlm.nih.gov/pubmed/?term=Gurbel P%5BAuthor%5D&cauthor=true&cauthor_uid=32129681). Synergistic influence of rivaroxaban on inflammation and coagulation biomarkers in patients with coronary artery disease and peripheral artery disease on aspirin therapy. [Future Cardiol.](https://www.ncbi.nlm.nih.gov/pubmed/?term=Synergistic+influence+of+rivaroxaban+on+inflammation+and+coagulation+biomarkers+in+patients+with+coronary+artery+disease+and+peripheral+artery+disease+on+aspirin+therapy) 2020 Mar;16(2):69-75.
- Combination Antiplatelet and Oral Anticoagulant Therapy in Patients With Coronary and Peripheral Artery Disease Focus on the COMPASS Trial. *Circulation.* 2019; 139:2170–2185.
- Mohamad A. Hussaina, Mark Wheatcrofta, Patrice Nault, Thomas F. Lindsay, Deepak L. Bhatt, Sonia S. Anand, Subodh Verma, and Mohammed Al-Omran. COMPASS for Vascular Surgeons: Practical Considerations. [Curr Opin Cardiol.](https://www.ncbi.nlm.nih.gov/pubmed/?term=COMPASS+for+Vascular+Surgeons%3A+Practical+Considerations) 2019 Mar;34(2):178-184.
- [Debus ES](https://www.ncbi.nlm.nih.gov/pubmed/?term=Debus ES%5BAuthor%5D&cauthor=true&cauthor_uid=32307302), [Nehler MR](https://www.ncbi.nlm.nih.gov/pubmed/?term=Nehler MR%5BAuthor%5D&cauthor=true&cauthor_uid=32307302); [executive committee of the Voyager PAD trial](https://www.ncbi.nlm.nih.gov/pubmed/?term=executive committee of the Voyager PAD trial%5BCorporate Author%5D). The Voyager PAD Trial - New Path for Post-revascularisation PAD Patients. [Eur J Vasc Endovasc Surg.](https://www.ncbi.nlm.nih.gov/pubmed/?term=The+Voyager+PAD+Trial+e+New+Path+for+Post-revascularisation+PAD+Patients) 2020 Apr 16. pii: S1078-5884(20)30276-8.
- Bonaca MP, Bauersachs RM, Anand SS, Debus ES, Nehler MR, Patel MR, Fanelli F, Capell WH, Diao L, Jaeger N, Hess CN, Pap AF, Kittelson JM, Gudz I, Mátyás L, Krievins DK, Diaz R, Brodmann M, Muehlhofer E, Haskell LP, Berkowitz SD, Hiatt WR. [Rivaroxaban in Peripheral Artery Disease after Revascularization.](https://www.ncbi.nlm.nih.gov/pubmed/32222135) N Engl J Med. 2020 Mar 28.
- *Hsin-Fu Lee, Yi-Hsin Chan and Chun-Li Wang. Using Rivaroxaban as Thrombolytic Treatment for a Patient of Pedal Arch Arterial Thrombosis with Suboptimal Result of Endovascular Therapy. Acta Cardiol Sin 2016; 32:623_626.*
- González-Fajardo JA, Ansuategui M, Romero C, Comanges A, Cases C, Gómez-Arbeláez D. Atrial Fibrillation and Surgical Patients with Peripheral Arterial Disease. Ann Vasc Surg. 2020 Mar 21. pii: S0890-5096(20)30236-3.
- Martin Björck, Jonothan J. Earnshaw, Stefan Acosta, Frederico Bastos Gonçalves, Frederic Cochennec, E.S. Debus, Robert Hinchliffe, Vincent Jongkind, Mark J.W. Koelemay, Gabor Menyhei, Alexei V. Svetlikov, Yamume Tshomba, Jos C. Van Den Berg. Editor's Choice-European Society for Vascular Surgery (ESVS) 2020 Clinical Practice Guidelines on the Management of Acute Limb Ischaemia. [Eur J Vasc Endovasc Surg.](https://www.ncbi.nlm.nih.gov/pubmed/?term=Editor’s+Choice+–+European+Society+for+Vascular+Surgery+(ESVS)+2020+Clinical+Practice+Guidelines+on+the+Management+of+Acute+Limb+Ischaemia)2020 Feb;59(2):173-218.

**Question 3**

Page 4, line 43 multiple comorbidities: The authors should consider whether the necessity of description the information about other important complications such as hypertension, diabetes and so on.

**Answer 3**

The patient, a former smoker, presented with frailty and multiple comorbidities, including multi-infarct dementia, previous recurrent transient ischemic attacks (TIA), chronic ischemic heart disease with three-vessel coronary artery disease deemed as non eligible for surgical intervention in 2013, Non-Valve Atrial Fibrillation (NVAF), dyslipidemia, chronic obstructive pulmonary disease (COPD), megaloblastic anemia, prior gastrectomy for gastric ulcer, and mild-to-moderate chronic kidney disease. The patient was not diabetic.

Drug therapy adopted was:

- dyslipidemia being treated with atorvastatin (20mg / day);

- arterial hypetension being treated with zofenopril (30mg / day), lercanidipine hydrochloride (10mg / day) and nitroglycerin in transdermal patches (5mg / day).

- megaloblastic anemia being treated with a supplement based on folate (acid (6S) -5- methyltetrahydrofolic), cyanocobalamin (vitamin B12), pyridoxine (vitamin B6), riboflavin (vitamin B2), betaine and zinc

- chronic obstructive pulmonary disease (BPCO) being treated with glycopyrronium bromide (one inhalation / day).

The patient was unsuitablenot suitable for myocardial (CAD) and peripheral (PAD) revascularization , given the remarkable severity and diffusion of atherosclerotic pathology.

**Question 4**

The author identified dyslipidemia as one of comorbidities in this case. However, the reviewer was not able to find out the information about parameters for dyslipidemia, such as LDL level. Please consider the necessity of the lipid data.

**Answer 4**

cholesterol and triglyceride parameters before using atorvastatin were:

total cholesterol: 250 mg / dl.

HDL cholesterol: 45 mg / dl.

LDL cholesterol: 181 mg / dl.

Triglycerides: 120 mg / dl.

after the use of atorvastatin the values have significantly decreased:

total cholesterol: 137 mg / dl.

HDL cholesterol: 57 mg / dl.

LDL cholesterol: 70 mg / dl.

Triglycerides: 51 mg / dl.

Question 5

The reviewer also suggest the necessity of the information about CHADS2 score (risk of cerebral infarction in patients with atrial fibrillation) and HAS-BLED score (risk of bleeding) in this case report.

**Answer 5**

The patient presents a CHADS2 score (risk of cerebral infarction in patients with atrial fibrillation) equal to 5 and HAS-BLED score (risk of bleeding) equal to 4

**Question 6.**

The authors should discuss about prognosis of critical lower limb ischemia with adequate references. The information would help us to understand the importance of this case report.

**Answer 6**

Success in treating critical limb ischemia is measured by amputation-free survival (AFS). Although patients with critical limb ischemia are revascularized by endovascular or surgical procedures, amputation rates remain high.

One year after the onset of critical limb ischemia approximately 25% of patients will have to undergo major limb amputation. While about 35-67% will experience major limb amputation within four years. Additionally, early post-operative mortality rates vary from 4% to 22% after major limb amputation. Numerous studies suggest that a polypharmacological approach is needed.

- anticoagulant or antithrombotic associated with anti Xa drugs (rivaroxaban) (fibrillating or non-fibrillating patients).

- antiplatelet agent (acetylsalicylic acid or clopidogrel) in associated coronary artery disease.

- Vasoactive (cilostazol), which plays an important role in the inhibition of proilferative cell replication processes also involved in the evolution of atherosclerosis and in the pathogenesis of restenosis.

These drugs certainly increase bleeding risk but not significantly if used at appropriate dosages. It is clear that this approach must always take into account the benefit / risk ratio and the degree of reversibility-irreversibility of the ischemic framework.


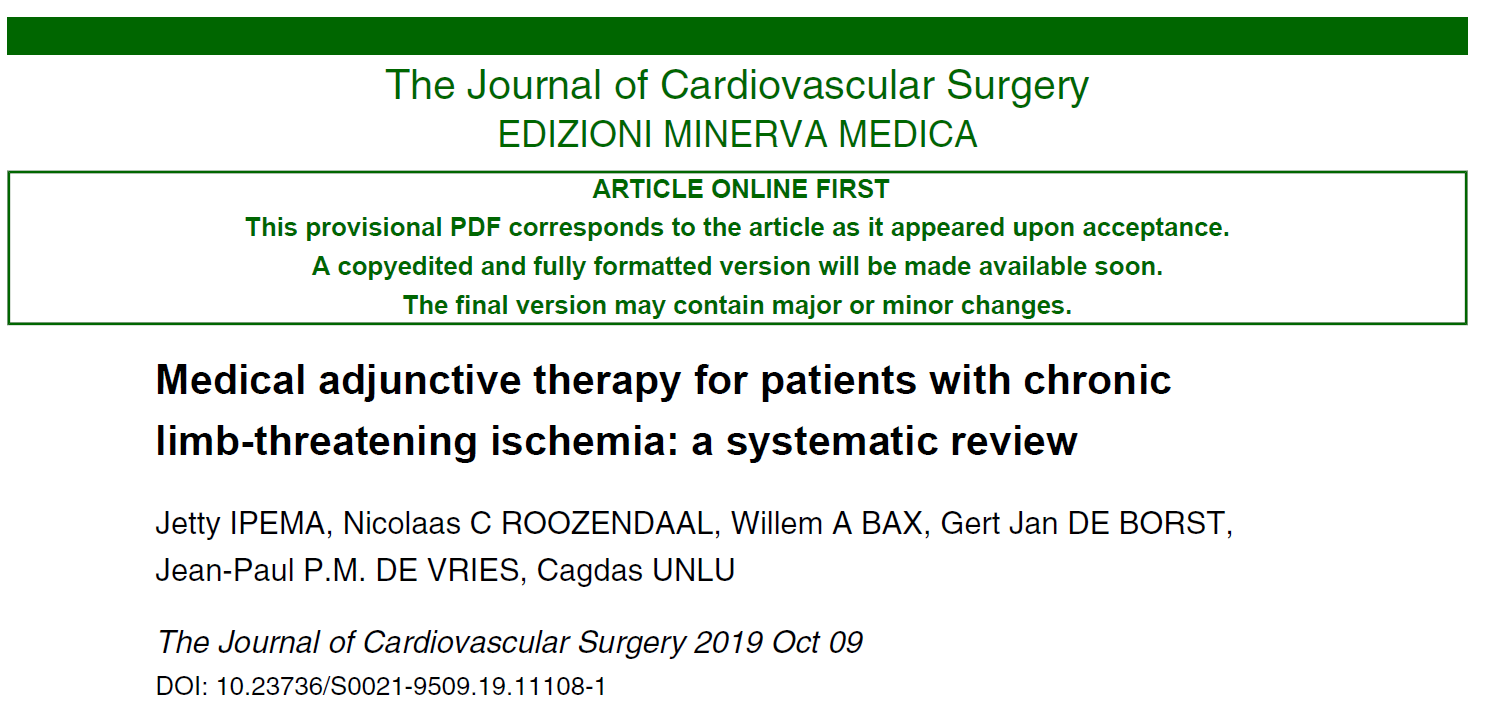


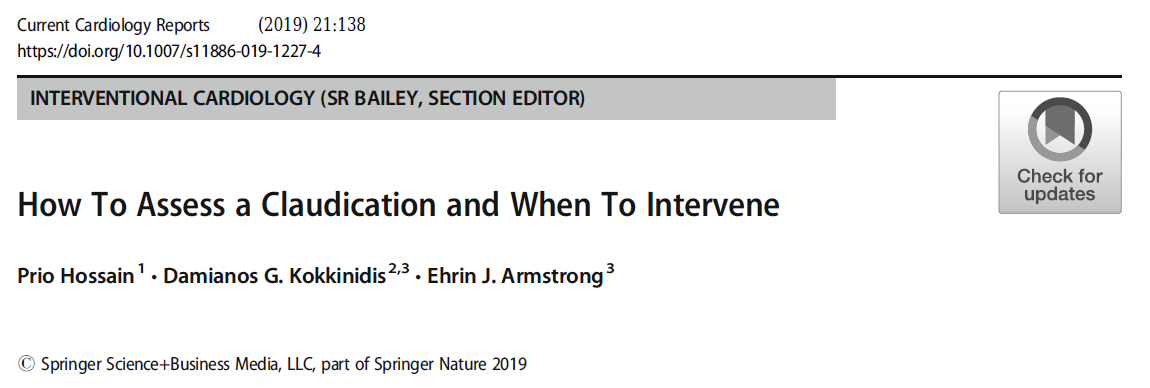


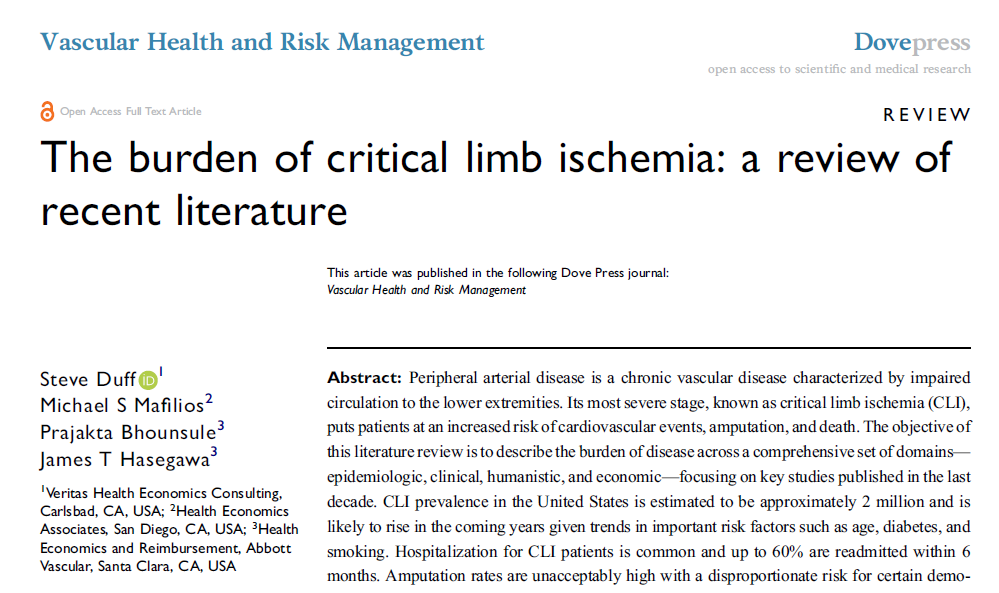


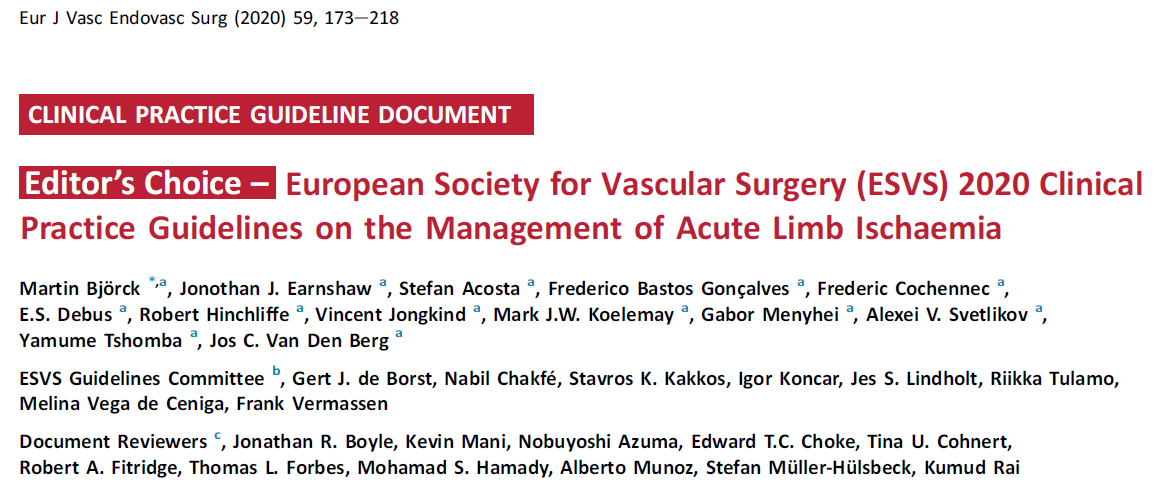


**Reviewer #2 - QUESTIONS**

Question 1

Anaswer 1

The ABI detected at the first evaluation was about 0.45 on the left and about 0.5 on the right and improved during the following evaluations until it was 0.6 on the left and about 0.65 on the right between 6 and 8 months from the start of therapy and then remained constant over time.

Laboratory parameters, except for the cholesterol levels that were reported, showed no substantial and significant changes during drug treatment.
